# Prediction of Lymphovascular and Perineural Invasion of Oral Squamous Cell Carcinoma by Combined Expression of p63 and Cyclin D1

**DOI:** 10.1055/s-0042-1760301

**Published:** 2023-01-30

**Authors:** Natheer H Al-Rawi, Sausan Al Kawas, Muwaffaq Al Ani, Ahmed Sameer Alnuaimi, Walid EL-Sayed, Mohammad S. Alrashdan

**Affiliations:** 1 Department of Oral and Craniofacial Health Sciences, College of Dental Medicine, University of Sharjah, United Arab Emirates; 2 Department of ENT, Tawam Hospital, Al-Ain, United Arab Emirates; 3 Primary Health Care Cooperation (PHCC), Doha, Qatar; 4 Department of Basic Medical and Dental Sciences, College of Dentistry, Gulf Medical University, Ajman, United Arab Emirates; 5 Department of Oral Biology, College of Dentistry, Suez Canal University, Ismailia, Egypt; 6 Department of Oral Medicine and Oral Surgery, Faculty of Dentistry, Jordan University of Science and Technology, Jordan

**Keywords:** p63, cyclin D1, lymphovascular invasion, perineural invasion, tissue microarray, oral squamous cell carcinoma.

## Abstract

**Objectives**  The aim of this study was to determine the value of immune expression of p63 and cyclin D1 in the prediction of lymphovascular invasion (LVI) and perineural invasion (PNI) in oral squamous cell carcinoma (OSCC).

**Materials and Methods**  Clinical and histopathologic features of 65 subjects with histologically confirmed OSCC were collected. Tissue microarray blocks representing all subjects were prepared for the immunohistochemical quantification of the nuclear expression of p63 and cyclin D1 using immune ratio plugin of image J software. Image analysis was performed by two independent pathologists. Independent samples *t* -test, analysis of variance, and receiver operating characteristic curve tests were used for statistical analysis. The level of significance was set at *p* ≤ 0.05.

**Results**  The optimum cutoff value for the prediction of LVI for p63 and cyclin D1 was found to be 100 and 93.2, respectively, while the optimum cutoff value for the prediction of PNI for p63 and cyclin D1 was found to be 95.9 and 94, respectively. p63 and cyclin D1 expression correlated with several clinicopathologic features of the studied population. p63 expression was a significant predictor of moderate/poorly differentiated OSCC compared with well-differentiated OSCC. A parallel combination of positive p63 and cyclin D1 increased the specificity of predicting LVI from 89.1% and 67.4% for either p63 or cyclin D1, respectively, to 93.5% with a positive predictive value of 92.5%. Similarly, the parallel combination of the two markers raised the specificity of predicting PNI from 70% and 77.5% for either p63 or cyclin D1, respectively, to 90% with a positive predictive value of 86.3%.

**Conclusion**  Combined overexpression of nuclear markers p63 and cyclin D1 can be considered as a valuable independent predictor of LVI and PNI, and hence tumor progression, in OSCC.

## Introduction

 Oral squamous cell carcinoma (OSCC) is the most common malignancy of the oral cavity, which accounts for the majority of head and neck cancers. According to the most recent report, the Global Cancer Incidence, Mortality and Prevalence database reported 377,713 new cases and 177,757 fatalities among males and females in 2020 due to OSCC. 1 Despite advancements in cancer detection and treatment, the 5-year survival rate for OSCC has not improved over the last two decades and is still around 60%. 2 3 Thus, it is critical to explore novel potential markers for risk assessment in OSCC. 

 A known feature of cancer is the dysregulation of the cell cycle machinery, and oral carcinogenesis is not an exception. 4 The transition from G1 to S stage in normal cell cycle is largely regulated by cyclin D1, a 45 kDa protein that was shown to be a critical factor in regulating cell proliferation, migration, and differentiation. Cyclin D1 has been extensively studied in the context of oral cancer and a remarkable connection was found between cyclins and oncogenesis. 5 Moreover, cyclin D1 gene amplification was shown to be prevalent in head and neck SCC. 6

 Cyclin D1 gene overexpression has been linked to several prognostic markers, including high T and N stages, advanced level, limited differentiation, and shorter survival rate. 7

 p63, located on 3q27–29 chromosome, is also an important gene in the cell cycle, differentiation, and apoptosis. 8 p63 was shown to be an ancestral member of the p53/p63/P73 gene family 9 10 and both p53 and p63 may play a role in malignant transformation. 11

 Prognosis and treatment decisions in OSCC are currently based on TNM staging, as determined by clinical examination, imaging studies, and histopathological features that are believed to be risk factors affecting patient outcomes. Other factors, such as the pattern of invasion of the tumor, the presence of perineural invasion (PNI), and the quality of the lymphocytic response, were shown to be statistically significant independent predictors of both local recurrence and overall survival. 12

 PNI is the consequence of a complicated interplay between invading tumor cells and the specific perineural habitat, which has been shown to alter outcomes in many malignancies. 13 Tumor cells infiltrating perineural tissues, following nerves, and/or encircling at least one-third of the nerve's diameter are common indicators of PNI in head and neck cancer. 14 PNI is observed in 5 to 90% of head and neck cancers. 13 Head and neck SCC patients with PNI have a much higher risk of experiencing poor outcomes and will need adjuvant treatment. 15

 Furthermore, tumor budding, depth of invasion, and lymphovascular invasion (LVI) have all been found to be predictors of lymph node metastasis (LNM) and prognosis in OSCC in several studies. 16 17 18 LVI is a pathological process in which tumor cells enter an endothelium-lined region of vascular or lymphatic vessels without causing damage to the underlying muscle walls. The penetration of tumor cells into lymphovascular spaces through the endothelial cell layer is a critical stage in the formation of tumor metastases, and it has been identified as a potential predictive trait in a variety of malignancies, including prostate cancer and colorectal cancer. 19 20 Previous literature showed that the presence of LNM in the original tumor is related to the detection of LVI, making it an important marker for disease progression in OSCC. 21

The aim of this study was to evaluate the significance of p63 and cyclin D1 as tumor markers in the prediction of LVI and PNI in OSCC.

## Materials and Methods

### Patients and Tissue Specimens

This retrospective study utilized the data of 65 individuals with OSCC from Tawam Hospital (Al-Ain, UAE). Tawam Hospital Ethical Committee (REC: AA/AJ/556) reviewed and approved all cases before the commencement of the study. Detailed clinicopathological information was gleaned from the patients' medical records. Two expert histopathologists (NH and SA) initially examined the hematoxylin and eosin (H&E)-stained tissue sections to confirm the histological diagnosis. The tumors originated from the tongue, floor of the mouth, cheek, gingiva, palate, or retromolar region. Since the vermilion boundary of the lip and the pharyngeal complex are not considered parts of the oral cavity, they were excluded. The history of tobacco and alcohol use was also retrieved from patients̀ records.

### Clinical and Histopathological Evaluation

American Joint Committee on Cancer Staging (AJCC) classification (sixth edition), pTNM level of the original tumor, amount of invasion, resection margin, PNI, LVI, and extra nodal growth were obtained from the histopathology reports of Tawam Hospital.

### Tissue Microarray

H&E slides from surgical specimens were examined by a pathology expert (NA). Initial H&E slides blocks that subsequently revealed a limited tumor area were deleted following comparison. Each tumor's invasive front was identified, and a donor paraffin block's core (0.5 cm) was punched out to perform the tissue microarray (TMA) blocks. There were 16 to 17 cores per block of TMA from the 65 samples put on four paraffin blocks.

### Immunohistochemistry

Paraffin sections were cut at 4 µm thickness, placed on positively charged slides, and dried in an oven for 30 minutes at 70°C. Deparaffinization, rehydration, and target retrieval were performed in the PT Link (Dako) using 3- in-1 procedure. Antibodies were detected using a visualization system (EnVision FLEX, Dako, K8000 Denmark) and 3, 3′-diaminobenzidine (DAB) chromogen at 25°C. Meyer's hematoxylin was used as a counterstain. Immunohistochemistry (IHC) staining for two antibodies, p63 (mouse anti p63 monoclonal antibody, clone 4A4, Dilution 1:100, Ventana Medical System, Tucson, Arizona, United States) and cyclin D1 (rabbit monoclonal antibody, clone SP4-R, Dilution 1:150, Ventana Medical system, Tucson, Arizona, United States), were performed on a Benchmark-ULTRA fully automated staining instrument (Roche Diagnostics, United States) using Ultra View Universal DAB Detection kit from Ventana. The antibodies were detected by DAB and then counterstained with Meyer's hematoxylin and bluing reagent.

### Image Acquisition

Olympus BX43 light microscope (Tokyo, Japan) was used to collect images in a bright field. A charge-coupled device color video camera (Olympus Life Sciences DP7, Tokyo, Japan) connected to a computer system was used to collect images with magnifications of x10, x40, and x100. DAB chromogen and hematoxylin were used to identify the tumor invasive fronts as the area of interest. All photographs were taken using the Olympus Cell Sense program software version 3.5.0 (Germany) installed on the computer system. The color density and white balance of all photographs were normalized before they were taken. To preserve the quality of the photographs, they were all saved in JPEG format.

### Image Analysis

**Fig. 1:**
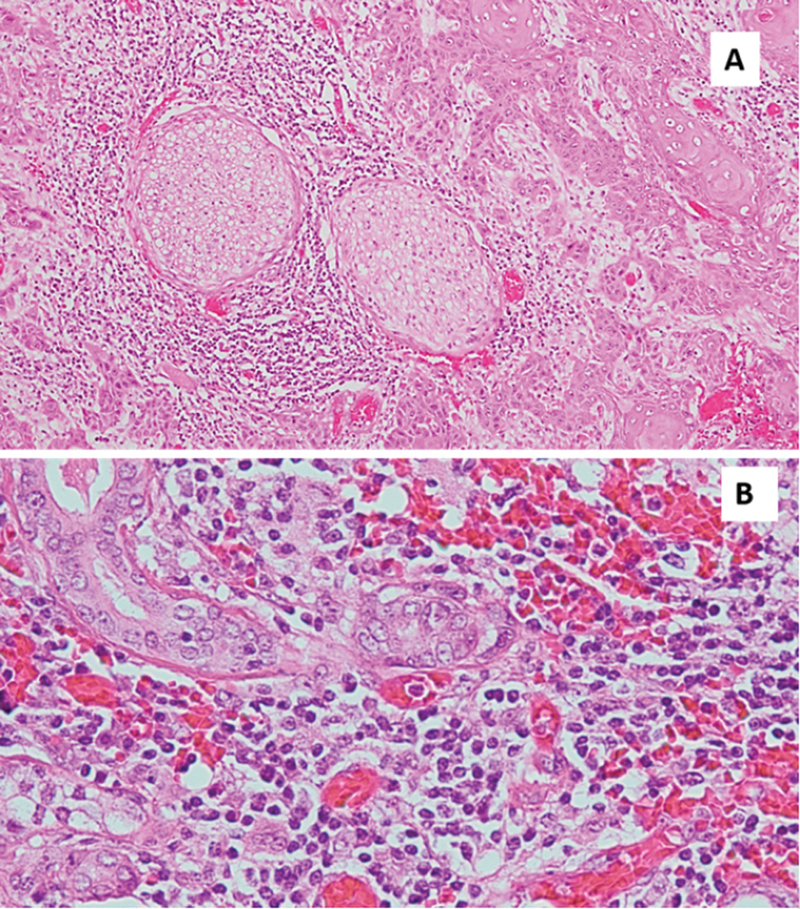
( **A** ) Perineural invasion of malignant epithelial cells (magnification X10). ( **B** ) Vascular invasion by malignant epithelial cells (magnification X40).

 The percentage of DAB-stained nuclear area to the total nuclear area was computed using the Immuno-Ratio plugin in Image J/Fiji after IHC staining for p63, and cyclin D1, using a standard protocol ( Fig. 1 ). The necrotic area of the specimen was not taken into account. 

 Two expert pathologists (NH and SA) independently verified the accuracy of Image J's analysis of the two biomarkers. A blinded histological diagnostic process was used for the quantification of every picture's immuno-score. Before beginning the study, both observers learned how to use Image J with the IHC profiler plugin and were able to maintain a consistent threshold level. 22

### Statistical Analysis

 SPSS IBM v.28 (Statistical Package for the Social Sciences, v28.0; IBM, Armonk, New York, United States) was used for the statistical analysis. The first step was to conduct a descriptive study. The independent samples *t* -test was used to examine the difference in mean values of quantitative continuous variables between two groups, whereas the analysis of variance test was used between multiple groups. Some chosen clinical and histopathological outcomes were compared with evaluate the prediction abilities of the examined markers using receiver operating characteristic (ROC) analysis. To evaluate an optimal cutoff value for the tested parameters, the shortest distance on the ROC curve was estimated at each one-half unit of the tested index. A distance on the ROC curve is equal to 



## Results

### Patients' Characteristics

 More than two-thirds of patients were men (46 vs. 19) and the age ranged from 28 to 91 years (mean 58.69 ± 14.34 years) ( Table 1 ). The tumor subsite was detected in the following sites: tongue ( *n*  = 41, 63.1%), buccal mucosa ( *n*  = 9, 13.8%), Jaw bones ( *n*  = 6, 9.2%), or multiple sites ( *n*  = 9, 13.8%). A smoking habit was reported in 16 patients (24.6%). According to the AJCC, T1 was detected in 22 (33.8%) patients, T2 in 15 (23.1%), T3 in 8 (12.3%), and T4 in 20 (30.8%) patients. pN0 classification was observed in 42(64.6%), pN1 in 6 (9.2%), pN2 in 13 (20%), and pN3 in 4 (6.2%) patients. According to overall AJCC disease stages, 15 (23.1%) had stage I, 8 (12.3%) had stage II, 12 (18.5%) had stage III, and finally, 30 (46.2%) had stage IV. Regarding the histopathologic degree of differentiation, 33 (50.8%) OSCCs were well-differentiated, 29 (44.6%) moderately, and 3 (4.6%) poorly differentiated. 

**Table 1:** Demographic and clinicopathologic characteristics of the study population

Gender	*n*	%	Age group (years)	*n*	%
Female	19	29.2	< 60	40	61.5
Male	46	70.8	60+	25	38.5
Total	65	100.0	Total	65	100.0
**Tobacco use**			**Comorbid condition**		
None	49	75.4	Hypertension	18	27.7
Cigarette smoking	5	7.7	Diabetes	20	30.8
Smokeless tobacco use	11	16.9	Cardiovascular diseases	5	7.7
Total	65	100.0	No comorbidity	42	64.6%
**Primary or recurrent cancer site**			**T stage (tumor size)**		
Tongue	41	63.1	T1	22	33.8
Cheek	9	13.8	T2	15	23.1
Jaws	6	9.2	T3	8	12.3
Multiple	9	13.8	T4	20	30.8
Total	65	100.0	Total	65	100.0
**Multiple vs. single tumor**			**Advanced T stage (T3-T4)**		
Single	53	81.5	Early T stage (T1–2)	37	56.9
Multiple	12	18.5	Advanced T stage (T3–4)	28	43.1
Total	65	100.0	Total	65	100.0
**N staging (cervical LN metastasis)**			**TNM stage**		
NO	42	64.6	Stage I	15	23.1
N1	6	9.2	Stage II	8	12.3
N2	13	20.0	Stage III	12	18.5
N3	4	6.2	Stage IV	30	46.2
Total	65	100.0	Total	65	100.0
**Cervical LN metastasis (N1–3)**			**Distant metastasis to the lung or liver**		
Negative	42	64.6	Negative	32	88.9
Positive	23	35.4	Positive	4	11.1
Total	65	100.0	Total	36	100.0
**Late-stage cancer (Stage 3–4)**			**Histological tumor grade**		
Early stage 1 2	23	35.4	G1: well differentiated	33	50.8
Late stage 3 4	42	64.6	G2: moderately differentiated	29	44.6
Total	65	100.0	G3: poorly differentiated	3	4.6
**Lymphovascular invasion**			**Perineural invasion**		
Negative	46	82.1	Negative	40	71.4
Positive	10	17.9	Positive	16	28.6
Total	56	100.0	Total	56	100.0

 Recurrent tumors were found in 14 cases (21.5%). Lymph nodes metastasis was found in 23 cases (35.4%). About two thirds of the cases had deep tumor invasion (>5mm). LVI was detected in only 10 cases (17.9%) and PNI was detected in 16 cases (28.6%). It is worth mentioning that all cases with LVI and PNI were associated with LNM ( Fig. 1 ). 

### Clinicopathological Characteristics of the Studied Sample

Table 2 shows the clinical characteristics of the study population. More than two-thirds of cases ( *n*  = 49) had deep tumor invasion. Evidence of residual tumors at resected margins was seen in 18 cases (27.7%). 

**Table 2:** Positive characteristics of the study population

Positive characteristics ( *n = 65)*	*n*	*%*
Deep tumor invasion (5+ mm)	49	75.4
Evidence of tumor at resected margins	18	27.7
Neck dissection is done along with tumor resection	46	70.8
Death from tumor or treatment's complications	6	9.2
Any end-point (loco-regional recurrence, cervical LN recurrence or death from tumor or treatment's complications, whichever earlier)	21	32.3

Abbreviation: LN, lymph node.

Forty-six cases underwent tumor surgical resection with neck dissection. One-third of the cases (32.3%) had treatment complications like loco-regional recurrence or cervical LN recurrence or death from tumor and/or treatment.

### Immunohistochemical Evaluation

**Fig. 2:**
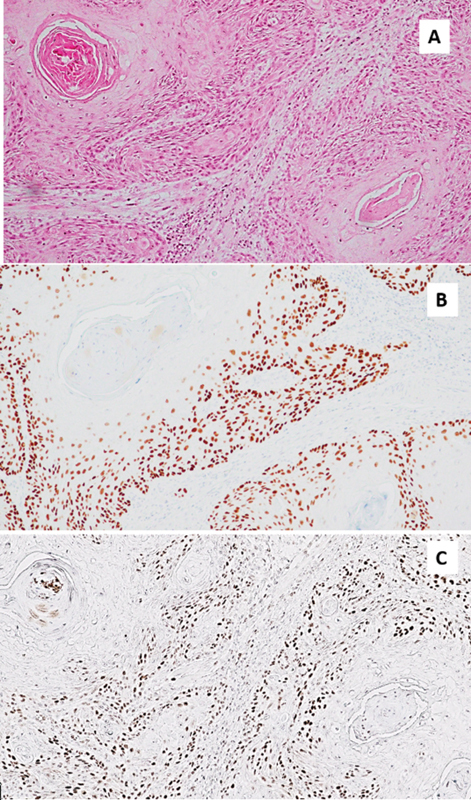
( **A** ) Hematoxylin and eosin atain of the selected area. ( **B** ) Nuclear expression of p63; strong expression of p63 at basal and suprabasal cells of malignant epithelial islands; nuclear expression of cyclin D1; strong expression of basal and suprabasal cells at the invasive front. (magnification x10).

 The IHC expression of the two studied markers was quantified and correlated with the clinicopathological characteristics of every subject as shown in Table 3 . Patients older than 60 years had a significantly higher expression of cyclin D1 marker compared with those younger than 60 years old. Higher expression of p63 was also identified in patients with distant metastasis compared with those not suffering from distant metastasis. Regarding histological grades, only moderately differentiated SCC expressed p63 in a significantly higher mean values when compared with patients having well-differentiated SCC ( Table 3 ). In addition, patients with recurrent tumors expressed a significantly higher mean tumoral p63, and tumoral cyclin D1 when compared with primary tumor patients ( Fig. 2 ). Positive PNI, LVI, or locoregional recurrence correlated with significantly higher expression of p63. 

**Table 3:** IHC expression of the studied markers

Variable	No.	p63 expression			Cyclin D1 expression		
		Mean	SD	*p* -Value	Mean	SD	*p* -Value
Female	19	66.3	31.2	0.48	81.7	24.6	0.13
Male	46	72.5	32.4		71.1	27.2	
< 60 years	40	64.9	32.7	0.06	66.9	28.7	0.002*
60+ years	25	79.9	29		86	18.3	
Cervical LN positive	42	67.9	34.1	0.31	74.1	26.4	0.95
Cervical LN negative	23	75.8	27.4		74.5	27.9	
Well differentiated	33	62.4	32.9	0.034*	69.6	30.2	0.16
Moderately/poorly differentiated	32	79.1	29		79	22.1	
No distant Metastasis	32	69.5	31.1	<0.001*	73.2	26.7	0.89
Distant metastasis	4	99.6	0.5		74.7	19.4	
Primary tumor	51	66.4	31.2	0.046*	69.7	27.5	<0.001*
Recurrent tumor	14	86.2	30.9		90.9	14.8	
End-point Negative	44	64.3	32.7	0.013*	60.9	32.8	0.52
End-point positive	21	83.9	26.4		67.1	37.1	
LVI negative	46	65.2	32.5	<0.001*	73.4	26.8	0.27
LVI positive	10	96.3	11.7		85.2	29.8	
PNI negative	46	64.3	34.3	0.003*	63.8	31.2	0.37
PNI positive	10	86.9	18.4		73.5	37.6	

Abbreviations: IHC, immunohistochemistry; LVI, lymphovascular invasion; PNI, perineural invasion; SD, standard deviation. ^*^ *p* <0.05.

 To test the predictive value of p63 and cyclin D1 for LVI and PNI (among other outcomes), only cases with an area under ROC curve more than or equal to 0.7 (associated with a fair test predictive power) were included for further analysis ( Table 4 ). p63 expression was a significant predictor of moderate/poorly differentiated tumors, specified end-points, LVI and PNI. 

**Table 4:** The predictive power (area under ROC curve) of the two tested markers when used to predict seven selected outcomes

Outcome	p63 (nuclear marker for cell mutation)-mean percent positive cells		Cyclin D1 (Nuclear marker of cell cycle/proliferation)-mean percent positive cells	
	AUROC	*p* -Value	AUROC	*p* -Value
Late-stage cancer (Stage 3–4) compared with early stage	0.53	0.72	0.51	0.92
Moderate/poorly differentiated tumor compared with well differentiated	0.67	0.022*	0.59	0.2
Deep tumor invasion (5+ mm)	0.54	0.67	0.66	0.05
Death from tumor or treatment's complications	0.62	0.34	0.65	0.22
Any end-point (loco-regional recurrence, cervical LN recurrence or death from tumor or treatment's complications, whichever earlier)	0.68	0.023*	0.56	0.42
Lymphovascular invasion under microscopic examination of tumor	0.89	<0.001*	0.72	0.09
Perineural invasion by tumor cells under histological examination	0.73	<0.001*	0.75	0.06

Abbreviation: AUROC, area under receiver operating characteristic curve **;** LN, lymph node.

NB: Lymphatic and vascular invasion were defined as the presence of tumor cell aggregates in endothelial-lined compartments without underlying muscle walls and invasion of the media of a vessel with intimal ulceration, respectively (ref Magi-Galluzzi). Perineural invasion was considered present when tumor cells were identified in the perineural space or epineurium (ref Rahima et al).

Magi-Galluzzi C, Evans AJ, Delahunt B, et al. International Society of Urological Pathology (ISUP) Consensus Conference on Handling and Staging of Radical Prostatectomy Specimens. Working group 3: extraprostatic extension, lymphovascular invasion and locally advanced disease. Mod Pathol 2011;24:26–38.

Rahima B, Shingaki S, Nagata M, Chikara S. Prognostic significance of perineural invasion in oral and oropharyngeal carcinoma. Oral Surg Oral Med Oral Pathol. 2004;97:423–31.

The optimum cutoff value for p63 was more than or equal to 100 and for cyclin D1 was more than or equal to 93.2 for predicting LVI. Similarly, the optimum cutoff value for p63 was 95.9 and cyclin D1 was 94 for predicting PNI.

 Parallel combination of the two parameters (i.e., considering a case as positive for LVI only when both p63 and cyclin D1 are positive) increased the specificity from 89.1% (for p63) to 93.5%. This translates to a marginal increase in positive predictive value from 89.2 to 92.5% ( Table 5 ). On the contrary, considering a case as positive when either of the two criteria is positive reduced the specificity from 89.1 (for p63) to 63% and the positive predictive value from 89.2 to 70.9%. 

**Table 5:** Validity parameters for the optimum cutoff value of two measurements when used alone or in combination to predict lymphovascular invasion

Predictors	Sensitivity	Specificity	Accuracy	PPV at pretest probability =		NPV at pretest probability =
				50%	90%	10%
Positive p63 (≥100) for predicting lymphovascular invasion by tumor cells	90.0	89.1	89.3	89.2	98.7	98.8
Positive cyclin D (≥93.2) for predicting lymphovascular invasion by tumor cells	80.0	67.4	69.6	71.0	95.7	96.8
Parallel combination of both p63 and cyclin D using both positive criteria for predicting lymphovascular invasion by tumor cells	80.0	93.5	91.1	92.5	99.1	97.7
Parallel combination of both p63 and cyclin D using either of the positive criteria for predicting lymphovascular invasion by tumor cells	90.0	63.0	67.9	70.9	95.6	98.3

Abbreviations: NPV, negative predictive value; PPV, positive predictive value.

 The cutoff values of p63 and cyclin D1, combined or alone, were also used to predict PNI ( Table 6 ). The parallel combination of the two parameters considering a case as positive for PNI only when both p63 and cyclin D are positive would increase the specificity from 70 (for p63) to 90%. This translates to a remarkable increase in positive predictive value from 67.6 to 86.2%. On the contrary, a parallel combination considering a case as positive when either of the two markers is positive reduced the specificity from 70 (for p63) to 57.5% and the positive predictive value from 67.6 to 63.8%. 

**Table 6:** Validity parameters for the optimum cutoff value of two measurements when used alone or in combination to predict perineural invasion

Predictors	Sensitivity	Specificity	Accuracy	PPV at pretest probability =		NPV at pretest probability =
				50%	90%	10%
Positive p63 (≥95.9) for predicting neural invasion by tumor cells	62.5	70.0	67.9	67.6	94.9	94.4
Positive cyclin D (≥94) for predicting neural invasion by tumor cells	75.0	77.5	76.8	76.9	96.8	96.5
Parallel combination of both p63 and cyclin D using both positive criteria for predicting neural invasion by tumor cells	62.5	90.0	82.1	86.2	98.3	95.6
Parallel combination of both p63 and cyclin D using either of the positive criteria for predicting neural invasion by tumor cells	75.0	57.5	62.5	63.8	94.1	95.4

Abbreviations: NPV, negative predictive value; PPV, positive predictive value.

## Discussion

 p63 is a p53-related DNA-binding protein that helps regulate differentiation and proliferation in epithelial progenitor cells. 24 Recently, p63 expression was shown to be a reliable indicator of histological grading and an early marker of poor prognosis in OSCC patients. Overexpression of p63 is known to occur in a subgroup of OSCC, with a substantial correlation to histological grade and survival rate. 25 Consequently, the examination of p63 pattern expression in OSCC may be a valuable and dependable molecular marker, deserving of any additional perceptive research. The current report shows that p63 expression correlates with several OSCC parameters including the histologic grading, staging, LVI, and PNI and can significantly predict both LVI and PNI. Correlation between the presence of perineural infiltration and the proportion of positively stained cells for p63 has been observed. Therefore, p63 expression may be effective for refining differentiation level, biological behavior, and perhaps OSCC diagnosis. 

 Cyclin D1, on the other hand, had some degree of positive predictive value for PNI but did not reach statistical significance ( *p*  = 0.06). When the combined positive expression of both p63 and cyclin D1 was used as a unified predictor of LVI and PNI, specificity measures and positive predictive value were markedly improved. 

 Among the various parameters used to predict the outcome of malignant disease, PNI is a widely used indicator of aggressive behavior, disease recurrence, morbidity, and death rate. 26 Despite its importance as a prognostic indicator, experimental studies to explore the molecular mechanisms responsible for PNI are limited. PNI is a form of metastatic tumor spread similar to but distinct from vascular or lymphatic invasion that hinders the ability to establish local control of malignancy because neoplastic cells can travel along nerve tracts far from the primary lesion and are often missed during surgery. 27 Most investigations have shown that PNI is, to different degrees, associated with disease recurrence, an increased probability of regional and distant metastasis, and an overall decrease in the 5-year survival rate. 28 29 30 A review by Woolgar cites evidence that OSCC exhibiting PNI in either major nerves or those of a smaller diameter (61 mm) are all associated with reduced survival rates and an increased risk of loco-regional recurrence. 31 In the present study PNI positively correlated with loco-regional recurrence but without reaching a significant statistical level. 

 Larsen et al. demonstrated that nodal involvement at the time of diagnosis of OSCC was significantly related to PNI (as well as grade, the presence of vascular invasion, and increasing tumor depth). 32 Furthermore, PNI was shown to correlate with late-stage disease and there is a strong tendency toward neural invasion in late-stage carcinoma. 33 34 However, in the present study, a non-significant statistical relation was observed between the two variables (PNI and late-stage disease) ( *r*  = 0.90; *p*  > 0.05). This could be attributed to the small sample size and the inclusion of different anatomical sites in the current report. 

 LVI is considered a critical step in locoregional spread and distant metastasis in several malignancies. 32 LVI is a pathologic process represented by the presence of tumor cells within definite endothelial-lined spaces, either lymphatic or blood vessels, as detected by H&E staining or IHC. 33 There is accumulating evidence that LVI stands as a negative prognostic factor in oral cancer patients with a higher risk of cervical LNM, loco-regional recurrence, and accordingly poorer prognosis. 34 LVI has been recently listed in the eighth AJCC staging system as an additional prognostic factor for OSCC; however, whether LVI represent an independent risk factor for recurrence or survival in OSCC is still questionable. 35

 A combination of PNI and LVI as prognostic indicators in OSCC was recently evaluated and justified by the fact that they both represent distinct pathologic features of the primary tumor that are related to tumor-microenvironmental interactions. 36 In their interesting investigation, Ting et al. reported that PNI/LVI double positive represents an independent predictor for LNM, distant metastasis, and poor survival in late-stage OSCC. 36

 The roles of p63 in multiple aspects of cancer, including tumorigenesis, cancer progression, and metastasis as well as how they impact other diseases are yet to be fully elucidated. The overexpression of p63 reflects the immaturity of the tumor cell lineage, which in turn may cause disruption of terminal differentiation and consequently preserve their ability to multiply 35 . Fisher et al demonstrated that p63 is critical to many aspects of cell signaling that serve a vast array of functions like cancer progression and metastasis. 9 Several reports showed that p63 represents an independent biomarker of OSCC aggressiveness and local advanced disease. 37 38 Cyclin D1 is another key player in cell cycle control. Cyclin D1 is known to form a complex with kinase 4/6 that in turn allows the cell to progress through the G1 phase by inactivating pRB through phosphorylation. 39 Disturbances in the function of cyclin D1 were shown to contribute to loss of normal cell cycle and hence tumorigenesis in OSCC. 40 Relevant literature reported a wide variety in the frequency of cyclin D1 overexpression (17.1–83%). 41 42

 Furthermore, cyclin D1 overexpression was postulated to represent an independent prognostic risk factor in OSCC as it showed significant associations with LNM, tumor cell differentiation, and tumor stage in some populations. 35

The routinely used tumor stratification based on TNM classification together with the histological grading alone is not sufficient to predict the individual prognosis of an OSCC. Therefore, there is an urgent need to establish additional prognostic factors based on a combination of IHC expression of some markers that are involved in tumor progression and antitumor immunity with some histological parameters that predict the tumor progression. Our novel approach of combining two tumor markers (p63 and cyclin D1) to predict LVI and PNI as critical indicators of disease progression in OSCC has shown promising results and will prompt further research into this field.

In conclusion, identifying a novel model of prediction of LVI and PNI from IHC expression of p63 and cyclin D1 was attempted using a statistical test that utilizes the parallel combination of the two markers for predicting a dichotomous outcome (LVI and PNI). The outcome was a significant improvement in the predictability and specificity of detecting LVI and PNI.

While the present study's findings are extremely encouraging, there are several caveats. To begin, a short follow-up revealed a small proportion of patients who developed locoregional recurrence and distant metastases. Second, a small percentage of PNI and LVI may necessitate a large sample size and additional testing.
